# Draft Genome Sequence of *Pseudomonas* sp. LS-2, Isolated from a Diseased *Gastrodia elata* Plant

**DOI:** 10.1128/MRA.01342-18

**Published:** 2018-11-21

**Authors:** Xiangyang Li, Xuefeng Zou, Kaishuang Zhang, Huaixin Meng, Dingxiang Shi

**Affiliations:** a College of Environment and Life Sciences, Kaili University, Kaili, People’s Republic of China; b Department of Environmental Science and Engineering, Fudan University, Shanghai, People’s Republic of China; Loyola University Chicago

## Abstract

We report here the draft genome sequence of Pseudomonas sp. LS-2, isolated from the decaying aerial stem of a Gastrodia elata plant. This genome harbored 96 potential genes implicated in bacterium-plant interactions, which may facilitate the adaptation of strain LS-2 to plant environments.

## Abstract (precis)

We report here the draft genome sequence of Pseudomonas sp. LS-2, isolated from the decaying aerial stem of a Gastrodia elata plant.

## ANNOUNCEMENT

Members of the genus Pseudomonas are Gram-negative bacteria that exhibit metabolic diversity and consequently are able to colonize a remarkable range of environmental conditions (1, 2). So far, Pseudomonas contains more than 190 defined species, some of which are pathogenic to plants, animals, and humans (1–3).

We collected a diseased Gastrodia elata plant from an experimental base (107°53′38″E, 26°31′44.40″N) at Kaili University. Then, Pseudomonas sp. LS-2 was isolated by spreading the decaying aerial stem of the diseased plant onto Luria-Bertani (LB) plates and culturing it at 30°C for 18 h. The 16S rRNA gene was amplified by a colony PCR using primers 27 F (5′-AGAGTTTGATCMTGGCTCAG-3′) and 1492 R (5′-TACGGYTACCTTGTTACGACTT-3′) and subsequently sequenced with an Applied Biosystems (ABI) 3730xl DNA analyzer. By performing a BLASTn analysis on the NCBI database, the 16S rRNA gene sequence has the highest possible similarity of 99% with that of Pseudomonas sp. 1-2. Therefore, strain LS-2 is assigned to the genus Pseudomonas.

The total DNA of strain LS-2 was extracted using the MiniBEST bacterial genomic DNA extraction kit (TaKaRa, Dalian, China) according to the manufacturer’s instructions. Sequencing of the strain LS-2 genome was conducted on an Illumina HiSeq 2500 platform with a paired-end 2 × 150-bp strategy. In total, 1,835,360,400 bp raw reads were generated, and 1,065,000,000 bp clean reads were obtained after removing reads containing adapters, reads containing poly-N (>10), duplicated reads (caused by the PCR during library construction), and low-quality reads (more than 40% with *Q* < 20 bases) using Trimmomatic (4) and FastUniq (5). These clean reads were assembled into 52 scaffolds using SOAPdenovo version 2.04 (6) and applying default parameters and a *k*-mer length of 77. The *N*_50_ and *N*_90_ scaffold sizes are 274,182 bp and 77,830 bp, respectively. The strain has a genome length of 6,868,005 bp with 155-fold coverage and an average GC content of 59.12%. The NCBI Prokaryotic Genome Annotation Pipeline (7) was used for genome annotation and identified 6,258 coding sequences, 67 RNA genes, and 286 pseudogenes in the strain’s genome. The genome of strain LS-2 harbored 96 potential virulence factors implicated in bacterium-plant interactions, such as flagella (8), the type III secretion system (T3SS) (9), type VI secretion system (T6SS) (8), and several effectors, like the hypersensitive response and pathogenicity (hrp) effector of T3SS (8) and hemolysin-coregulated protein (hcp) effectors of T6SS (10).

As of June 11, 2017, there were genome sequences for more than 3,800 Pseudomonas strains, representative of 167 species, publicly available in the NCBI genome database. Based on average nucleotide identity (ANI) analysis using JSpecies (11), Pseudomonas sp. LS-2 has a maximum ANIm (ANI calculated by MUMmer) of 86.8% with Pseudomonas abietaniphila KF717 (GenBank accession number BBQR00000000). This value is lower than the proposed species boundary ANI cutoff of 95 to 96% (11). According to the closest match in the NCBI database, this genome may represent a new Pseudomonas species.

### Data availability.

This whole-genome shotgun project has been deposited at DDBJ/ENA/GenBank under the accession number QXTJ00000000. The version described in this paper is version QXTJ01000000. The corresponding raw sequencing data sets have been registered in the NCBI Sequence Read Archive under the accession number SRR7892667.
